# Management of Monosymptomatic Nocturnal Enuresis in Children: A Pilot Randomised Control Trial of Body‐Worn Versus Bell‐and‐Pad Alarm Device

**DOI:** 10.1111/jpc.70543

**Published:** 2026-08-25

**Authors:** Blake Peck, Daniel Terry, Bronwyn Peck, Andrea Green, Kerrie Svedas, Shirley Whitaker, Monica Harrop, Erica Pearce, Mark Nethercote, Natasha Burgess, Ha N. D. Le, Sithara Wanni Arachchige Dona, Susan Gibb

**Affiliations:** ^1^ School of Nursing and Midwifery, Faculty of Health Deakin University Burwood Australia; ^2^ School of Nursing and Midwifery University of Southern Queensland Ipswich Australia; ^3^ Grampians Regional Continence Service, Ballarat Health Services Ballarat Australia; ^4^ Barwon Health Geelong Australia; ^5^ The Royal Children's Hospital Melbourne Australia; ^6^ Deakin Health Economics, School of Health and Social Development, Faculty of Health, Institute for Health Transformation Deakin University Melbourne Australia

**Keywords:** alarm intervention, bedwetting, children, enuresis, monosymptomatic

## Abstract

**Aim:**

To compare bell‐and‐pad alarm therapy to body‐worn alarm therapy for the management of monosymptomatic enuresis in children 6–16 years of age in Victoria, Australia.

**Method:**

A sample of 76 children with monosymptomatic enuresis were randomly allocated to a control group (bell‐and‐pad alarm, *n* = 38) or an experimental group (body‐worn alarm, *n* = 38). All children were treated for 8 weeks. Children who had not been successful, defined as 14 consecutive dry nights, continued therapy up to 16 weeks. The two alarm types were compared using dichotomous cross‐tabulations and chi‐squared tests of independence based on the most positive outcome versus the other outcomes. The costs of the treatment were calculated from the service provider's and family's perspective. Costs were in 2023 Australian dollars.

**Results:**

Treatment success was achieved in 22 children (68.7%) in the body‐worn alarm group and 34 children (94.4%) in the routine (bell‐and‐pad) group (*p* = 0.010). The bell‐and‐pad alarm also performed better on three other indicators: Alarm used every night (*p* = 0.018), alarm woke parent (*p* = 0.011), and child was compliant with alarm use (*p* = 0.015). The body‐worn treatment was found to be cheaper than the bell‐and‐pad treatment ($598 per child) but was not as effective.

**Conclusion:**

The success rates for treatment were high in both groups but the bell‐and‐pad alarm was found to be more effective than the body‐worn alarm used in this study. Further research is recommended, with a larger population, to establish more definitive support for clinical decision making.

**Trial Registration:** Australian and New Zealand Clinical Trials Registry ACTRN12619000354189

## Brief Points

- What is already known on the topic
  ○ Alarm therapy is regarded as the first line therapy for children with monosymptomatic enuresis.
  ○ Therapeutic outcomes are improved when the child and family are motivated.
  ○ Waiting times for alarm therapy in continence services within Victoria, Australia are commonly 6–12 months.
- What this paper adds
  ○ The bell‐and‐pad alarm is the more effective modality.
  ○ The body‐worn alarm which is more affordable and accessible had an efficacy of 68.7%.
  ○ These findings support a model where bell‐and‐pad alarm is offered if body‐worn alarm treatment is not successful.

## 1 Introduction

Bedwetting, or enuresis, is defined as involuntary urination during periods of sleep at the age of 5 years or older [1]. Bedwetting is a common chronic childhood condition with approximately 10% of 7‐year‐olds affected, representing a significant burden to children and families worldwide [2]. Enuresis can be monosymptomatic, where there is no associated lower urinary tract dysfunction (urinary frequency, urgency and/or daytime wetting) or non‐monosymptomatic enuresis where incontinence during sleep occurs in combination with other bladder symptoms [3].

Enuresis is a distressing condition that has been shown to have an impact on a child's psychosocial wellbeing [4] with up to 30% of children fulfilling the criteria for the Diagnostic and Statistical Manual of Mental Disorders IV psychiatric disorders [5]. Research suggests self‐esteem, emotional wellbeing and relationships with family and friends are significantly lower in those with enuresis than in healthy peer controls [6, 7]. These impacts on a child's well‐being underscore the importance of access to timely therapy [8].

There are two established first‐line treatments for nocturnal enuresis: alarm therapy and pharmacological therapy (desmopressin). In a Cochrane review inclusive of 74 trials, it was unclear if alarm therapy is more effective than desmopressin, recognising however, there is likely to be a lower risk of adverse events with alarms than with desmopressin [9].

The bell‐and‐pad (BP) alarm system is widely used and regarded by many clinicians as the preferred treatment for managing nocturnal enuresis in childhood in Australia; as such, it is routine practice to use alarm therapy first when indicated and to only consider combination therapy in treatment resistant cases [10, 11]. BP systems are, however, expensive to purchase, and it is challenging for health services to make these devices readily available, resulting in prolonged waiting periods (upwards of 6 months). At a comparative cost ratio of approximately 1:20 per unit (BP being 20 times more expensive), the body‐worn (BW) alarm offers a potential readily accessible and economically viable alternative.

There are no contemporary randomised controlled trials comparing the effectiveness of the two devices for enuresis treatment. While it is appreciated optimal clinical outcomes in overcoming enuresis are achieved if therapy is commenced as soon as a child is psychologically ready to begin [4, 12], the lack of timely access to an affordable and evidence‐based therapeutic modality poses a conundrum.

The purpose of this study was to prospectively compare the efficacy and costs of BP alarm therapy to BW alarm therapy for the management of monosymptomatic enuresis in children 6–16 years of age.

## 2 Method

A prospective, randomised and adaptive clinical control trial to evaluate the efficacy of two alarm devices for the management of monosymptomatic enuresis in children 6–16 years of age was conducted (clinical trial registration: ACTRN12619000354189, https://www.anzctr.org.au/Default.aspx). Data were collected from July 2019 to July 2022 in Victoria, Australia. The study setting was five specialist continence services in Victoria, Australia, inclusive of regional (*n* = 4) and metropolitan services (*n* = 1). Data collection was divided into three phases: Initial Clinical Assessment of the child (T1), Interim Clinical Assessment of the child (8 weeks, T2) and the Final Clinical Assessment of the child (T3) for children who have not achieved dryness at T2 (Table 1).

**TABLE 1:** Three phases of data collection.

	T1 (Week 1)	T2 (8 weeks)	T3 (16 weeks)
Part A^a^	X		
Economic questionnaire^b^	X	X	X
CHU9D^c^	X	X	X
Part B^d^		X	X

*Note:* If the child had not achieved dryness at T2 a further 8 weeks of treatment was initiated, and the Final Clinical Assessment of the child (T3) occurred following 16 weeks of treatment.

^a^ Initial 13 questions of a two‐part 27‐item instrument developed for this study (the questionnaire was not subject to formal validation) to determine eligibility and the child's enuresis history, co‐morbid conditions and any medication use.

^b^ Eight questions associated with the economic implications of health service use and costs associated with treatment for the child's bedwetting: number of visits to health professionals (urologists, paediatricians, general practitioners, psychiatrists and continence nurses), additional expenses (pull‐up pants, bed sheets and additional washing activities).

^c^ Child Health Utility Nine Dimensions—validated tool for measuring children's health‐related quality of life [13]. A single utility score for each individual was generated using the Australian values [14].

^d^ Further 14 questions of a two‐part 27‐item instrument developed for this study (the questionnaire was not subject to formal validation) to determine primary and secondary outcome measures (dryness for 14 nights, duration of alarm therapy, relapse, alarm characteristics and response of child to the therapy).

Inclusion criteria were (1) aged 6–16 years of age, (2) had not previously used an enuresis alarm and (3) had no daytime urinary symptoms of or co‐morbid constipation/faecal incontinence. Children were excluded if they had a diagnosis of a neurodevelopmental concern or were using adjunctive pharmacotherapy (anticholinergics, beta‐3 agonist, synthetic vasopressin or other). At initial screening, children found to have non‐monosymptomatic nocturnal enuresis (NMNE) were treated prior to being recruited to the alarm therapy [1, 3]. As the study was of unselected patients with monosymptomatic enuresis randomised to two alarm types, it was reasonably expected that any nocturnal polyuria would be evenly distributed between the two groups.

Study approval was achieved through each participating institutional Human Research Ethics Committee and in adherence with the ethical principles for medical research on human beings set out in the Declaration of Helsinki (approval number: LNR/15/BHSSJOG/1). All participants, including the children, provided written informed consent/assent.

Sample size was calculated to be 252 patients based on 0.8 power of non‐inferiority [15]. The intention was to enrol 278 patients into the study to accommodate 10% of all participants being lost to follow‐up.

The primary outcome measure was achieving dryness for at least 14 consecutive nights, consistent with ICCS guidelines [1]. All children were initially treated for 8 weeks, but those who had not achieved dryness continued treatment for up to 16 weeks when final outcomes were evaluated. Treatment failure was defined as not achieving 14 consecutive dry nights. Secondary outcome measures were seven other categorical indicators, three quantitative duration measures, the child's quality of life and the health services and additional resources that families used at baseline and follow up. The devices used were the P Model BP enuresis alarms (Ramsey‐Coote Instruments, Australia) and the M04 ultimate (BW) enuresis alarm (Malem Medical, the United Kingdom).

Children recruited were randomly allocated to the BW or the BP alarm device. The allocation was not determined by a formal algorithmic randomisation schedule but rather emerged naturally through the probabilistic nature of participant arrivals during the period when both device types were concurrently available in the service. Specifically, as eligible children presented to the service, they were assigned to whichever device was next available, resulting in a de facto randomisation process shaped by timing and availability rather than predetermined assignment.

An alarm device was allocated to each family, and staff provided a demonstration and instructions on the use of the specific device. They were provided with a ‘Bed Wetting Diary’ to record information for each day of the therapy. This included the wet/dry status of the child overnight, the time of the alarm, the action taken by the child and parent in response to the alarm, as well as any overnight voiding (without alarm).

The economic evaluation of the intervention was conducted from the service provider and family's perspectives. We included all the costs associated with the delivery of the treatment programme. As the BW devices were new to staff, there were some initial training costs involved. Overhead costs to reflect superannuation, building and facilities, etc. were estimated as 20% of programme costs. Resources and unit cost data were obtained from the trial records. Programme costs were actual expenses related to programme delivery, valued in 2023 Australian dollars (AUD). Costs collected before 2023 were converted to 2023 AUD using the consumer price index reported in the ABS [16].

Programme costs were calculated by multiplying the unit cost with the resources used for both BW experimental treatment and the BP alarm (i.e., usual treatment). It was assumed that children with BP alarms required the same nurse and GP time. Programme cost per child was calculated by dividing the total cost by the number of children.

The incremental cost‐effectiveness ratio (ICER) was calculated by dividing the incremental costs (in Australian dollars) by the incremental benefits (in their natural units, e.g., utility scores for children's HRQOL). The incremental cost/effectiveness of the programme was calculated as the difference (per child) in the average costs/effectiveness (i.e., dryness for more than 14 days or child utility scores) to the service provider between the BW alarm treatment and the comparison group (i.e., BP alarm).

### 2.1 Data Analysis

Data were imported to SPSS (Statistical Package for Social Sciences Chicago, IL USA) software, version 25, for analysis. The two types of devices (BP and BW) were compared with respect to the categorical variables (Table 2) using dichotomous cross‐tabulations and chi‐squared (*χ* ^2^) tests of independence based on the most positive outcome versus the other outcomes. For those children who attained dryness for ≥ 14 nights, non‐parametric Mann–Whitney U test was used to compare the two types of devices with respect to two measures of duration. Similarly, Mann–Whitney *U* test was conducted to investigate HRQoL difference between the two treatment groups at baseline and follow‐up surveys as HRQoL data was not normally distributed (Table 3). Significance was determined at *p* ≤ 0.5.

**TABLE 2:** Group characteristics.

Variable	*n*	BP	*n*	BW	*p*
Mean age (SD)	38	7.6 (1.5)	38	7.4 (1.4)	0.244
Sex ratio (males to females)	38	0.31	38	0.21	0.238
Treatment length (days)	38	61.8	38	74.1	0.156

*p* < 0.5, or *p* < 0.01 statistically significant.

**TABLE 3:** Pre‐ and post‐treatment health related quality of life (HRQoL) outcomes for each device.

Variable	*n*	Pre‐treatment mean	Post‐treatment mean	*p*
Bell‐and‐pad	35	0.969	0.056	0.007
Body‐worn	31	0.952	0.061	0.010

*p* < 0.5, or *p* < 0.01 statistically significant.

In the bivariate analysis, as the total cost was not normally distributed, the Mann–Whitney U test was used to explore the difference in health service costs between the two treatment groups at baseline and follow‐up survey at discharge [17]. Analyses were conducted using STATA 18.0 [18].

## 3 Results

Due to recruitment difficulties during the COVID‐19 pandemic, we were unable to achieve the original intended sample size. Of the 115 children assessed for the study, 76 children with enuresis were ultimately enrolled into the study. Their characteristics were previously presented in Table 2. Adjunctive pharmacotherapy (synthetic vasopressin) had been used amongst three children for 3 months or less, which had been ceased as a therapy prior to recruitment.

Thirty‐four of 36 children using the BP attained dryness for ≥ 14 nights compared with 22/32 children using the BW device, noting five were lost to follow‐up within this group (Figure 1). The BP device also produced higher proportions of positive outcome measures for six of the secondary outcomes. The BW device performed better on only one outcome, waking the child, but this was not significantly different to the result for the BP (Table 4).

**TABLE 4:** Primary and secondary outcomes.

Variable	Affirmative response to question	*n*		Percentage of affirmative response to question category		*p*
		BP (*n* = 36)	BW^a^ (*n* = 32)	BP	BW	
The child was dry ≥ 14 nights	Yes	34	22	94.4	71.0	0.010**
Relapse	No	33	25	94.3	83.3	0.282
Alarm used every night	Yes	35	25	97.2	78.1	0.018*
Alarm awoke the child	Yes	35	32	97.2	100.0	0.619
Alarm awoke the parents	Yes	36	26	100.0	71.2	0.011**
False (positive) alarms	No	30	19	85.7	59.4	0.075
Child turned alarm off and went back to sleep	No	30	22	85.7	68.7	0.174
False (negative) non‐alarms	No	28	22	80.0	68.7	0.461

^a^ Five lost to follow‐up.

* *p* < 0.5.

** *p* < 0.01.

**FIGURE 1:**
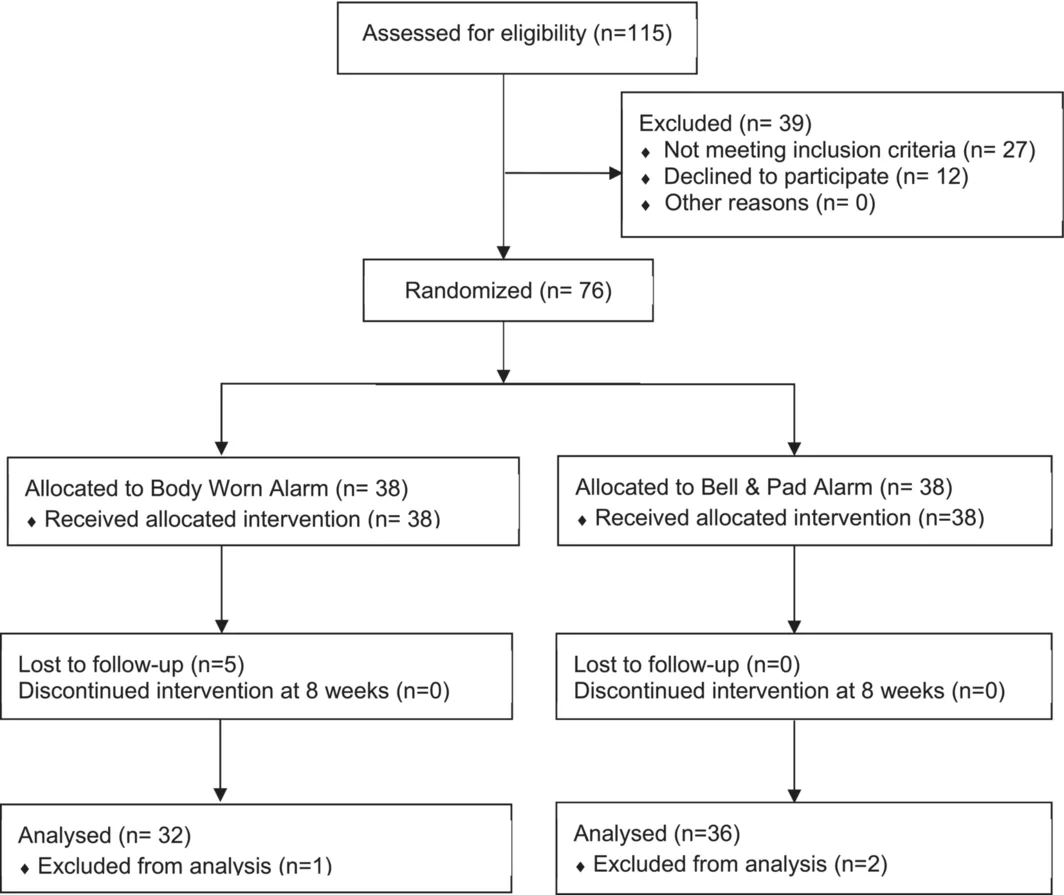
Flow diagram of patients who accepted to enter the study at a Specialist Continence Service, and the random allocation to intervention and control groups.

For those children who attained dryness for ≥ 14 nights, there was no difference in the duration of alarm use or the time to dryness between devices (Table 5).

**TABLE 5:** Additional outcomes.

Variable	BP		BW^a^		*p*
	*n*	Mean	*n*	Mean	
Duration of use (nights)	33	28.05	28	34.48	0.158
Nights to attain dryness	21	29.55	28	21.59	0.053
Children's HRQOL baseline	35	0.969	31	0.952	0.230
Change in HRQoL at follow‐up	35	0.056	31	0.061	0.688

^a^ Five lost to follow‐up.

*p* < 0.5, or *p* < 0.01 statistically significant.

Children in both groups demonstrated improved HRQoL post‐treatment within the 3‐months intervention (Table 3) and this was not significantly different between the groups.

The average overall cost of the BW treatment per child was $211 compared with $810 for BP treatment. The incremental cost of the BW and BP treatment was −$598 (Table 6).

**TABLE 6:** Programme costs during the intervention (AUD, 2023).

Intervention costs	Cost items	BW (AUD2023)	BP (AUD2023)
Team development	Training for nurses	$801	$0
Staffing costs	Nurse time	$4064	$4064
	GP time	$120	$120
	Administrative staff	$570	$495
Programme resources	Alarms (for 10 devices)	$1608	$18 000
Overhead cost		$1433	$4536
Total costs		$8026	$26 720
Number of children		38	33
Cost per child		$211	$810

*Note:* Assumed that children with usual treatment (i.e., Bell‐and‐pad) required the same amount of nurse and GP time as those in the body‐worn treatment. Costs were estimated in 2023 Australian dollars and were rounded to the nearest dollars. The overhead cost is a 20% of the total cost of each treatment arm.

There was no difference in the family cost between children in BW and the BP treatment (*p* = 0.644) at baseline. At discharge, the information on health service use for those in BP treatment was not reported (Table 7).

**TABLE 7:** Health service use cost to family.

Service costs items	Baseline (AUD2023)		Discharge (AUD2023)	
	BW mean (SD)	BP mean (SD)	BW mean (SD)	BP mean (SD)
Total health services expenses per child	$108 (310)	$26 (98)	$54 (253)	$0
Additional expenses	$61 (41)	$50 (25)	$84 (100)	$28 (51)

*Note:* The mean and standard deviation (SD) were calculated based on the number of patients who reported. Health service use at the discharge for BP was not reported. We assumed families did not use additional services and therefore did not incur additional cost.

There was also no statistically significant change between treatment groups at both pre‐ (*p* = 0.731) and post‐treatment (*p* = 0.132). The BW was cheaper but not more effective than the BP (Table 8).

**TABLE 8:** Cost‐effectiveness of body‐worn compared with bell and pad.

Outcome	Body‐worn	Bell and Pad	Mean difference in outcomes between the two groups	ICER (Incremental cost‐effectiveness ratio)
Mean cost of the programme per child	$211	$810	‐$598	
Dry ≥ 14 nights (mean)	16.89	24.19	−7.30	Dominated
Quality of life (mean)	0.95	0.97	−0.02	Dominated

*Note:* Dominated indicates that the body‐worn is cheaper but is not effective and this is not cost effective.

## 4 Discussion

Despite the limitations of sample size, the BP device achieved more positive outcomes than the BW device. With respect to the primary outcome measure (achieving dryness for 14 consecutive nights), the BP device yielded a 94.4% versus a 68.7% success rate for the BW device. The efficacy rate of the BP device is higher than the 65% success rate reported as the expected clinical standard when employing the enuresis alarm with a typical population [12, 19]. This may reflect this population being highly selected for MNE.

There are several potential reasons for these observed differences in efficacy. The cornerstone of alarm therapy success is the ability of the device to wake the child and parent [1]. The process of getting out of bed conditions the child to wake and void in the toilet with a full bladder and is part of the training and supports reinforcement [13]. The use of the BP device each night (*p* = 0.018) would have increased the opportunities for reinforcement, and we suggest that the design of the BP device always being on the bed likely helped to achieve this.

In addition, the BP device was superior to the BW device regarding waking the parent (*p* = 0.011). This would have enabled the parent to assist with the resetting of the alarm and ensuring the child woke completely and visited the bathroom, which may have contributed to greater success.

The BW device did wake the child more often in this study and this may be due to its proximity to the child. This difference was not significant, but it is an area worthy of more exploration. Different alarm devices have the capacity for variation in alarm volume and mechanisms to turn the device off, so our results may not be generalised necessarily to all other BW alarm devices.

Children's health‐related quality of life improved in both treatment groups. These changes are clinically meaningful within the very short‐term (< 3‐month intervention) [14]. This lends support for timely treatment for enuresis even with a less efficacious device if that is what is available. However, despite the BW device being less expensive, it is less effective and thus not cost‐effective compared with the BP device as shown in the ICER analysis. This analysis compares the additional cost of one intervention relative to another against the additional health benefit it provides. Overall, what is found is that it reinforces the importance of balancing affordability with clinical efficacy when selecting enuresis treatments.

Nevertheless, while the BW device was found to underperform against the primary and some secondary variables, it does offer an opportunity for children, families and clinicians. Research has highlighted the merit of capitalising on familial and child motivation when considering alarm therapy [20]. Within Victoria, it is common to wait 6–12 months to access enuresis expertise, which impacts negatively on therapeutic outcomes. We suggest therefore that general practitioners and paediatricians alike consider readily accessible and cost‐effective BW devices as a first‐line approach to treatment. Our study suggests that just less than 70% of children with monosymptomatic enuresis who receive treatment with a BW device will succeed, and those who fail treatment can progress to BP therapy. Irrespective of the device being used, the HRQoL implications are tangible for child wellbeing.

This study was not powered as a non‐inferiority study and therefore could not conclusively demonstrate differences between the results for the BP and BW devices. Future studies comparing the BP device with a BW device with a larger sample size are required.

## 5 Conclusions

Our findings from this pilot suggest that the BP device, whilst more costly, was more effective in achieving dryness in children with monosymptomatic enuresis for ≥ 14 nights—the primary outcome measure—when compared with the BW device. Treatment with either device was associated with an improved health‐related quality of life in children.

## Funding

The authors have nothing to report.

## Ethics Statement

Study approval was achieved through the participating institutional Human Research Ethics Committee and in adherence with the ethical principles for medical research on human beings set out in the Declaration of Helsinki (approval number: LNR/15/BHSSJOG/1).

## Consent

All participants, including the children, provided written informed consent/ascent.

## Conflicts of Interest

The authors declare no conflicts of interest.

## Supporting information


**Data S1:** CONSORT 2025 checklist item description.

## Data Availability

The data that support the findings of this study are available from the corresponding author upon reasonable request.

## References

[1] T. Nevéus , E. Fonseca , I. Franco , et al., “Management and Treatment of Nocturnal Enuresis—An Updated Standardization Document From the International Children's Continence Society,” Journal of Pediatric Urology 16, no. 1 (2020): 10–19. 10.1016/j.jpurol.2019.12.020 32278657

[2] A. J. Wright , “The Epidemiology of Childhood Incontinence,” in Pediatric Incontinence: Evaluation and Clinical Management (Wiley, 2015), 37–60.

[3] National Institute for Health and Care Excellence , Bedwetting in Under 19s (Clinical Guideline CG111) (National Institute for Health and Care Excellence, 2010), https://www.nice.org.uk/guidance/cg111. 39808006

[4] P. H. Caldwell , A. V. Deshpande , and A. Von Gontard , “Management of Nocturnal Enuresis,” BMJ 347 (2013): f6259. 10.1136/bmj.f6259 24170156

[5] A. Von Gontard , D. Baeyens , E. Van Hoecke , W. J. Warzak , and C. Bachmann , “Psychological and Psychiatric Issues in Urinary and Fecal Incontinence,” Journal of Urology 185, no. 4 (2011): 1432–1437. 10.1016/j.juro.2010.11.051 21349549

[6] B. Iscan and N. Ozkayın , “Evaluation of Health‐Related Quality of Life and Affecting Factors in Child With Enuresis,” Journal of Pediatric Urology 16, no. 2 (2020): 195.e1–195.e7. 10.1016/j.jpurol.2019.12.018 32008988

[7] I. Jönson Ring , T. Nevéus , A. Markström , K. Arnrup , and F. Bazargani , “Nocturnal Enuresis Impaired Children's Quality of Life and Friendships,” Acta Paediatrica 106, no. 5 (2017): 806–811. 10.1111/apa.13787 28199734

[8] J. Vande Walle , S. Rittig , S. Bauer , P. Eggert , D. Marschall‐Kehrel , and S. Tekgul , “Practical Consensus Guidelines for the Management of Enuresis,” European Journal of Pediatrics 171, no. 6 (2012): 971–983. 10.1007/s00431-012-1687-7 PMC3357467 22362256

[9] P. H. Y. Caldwell , M. Codarini , F. Stewart , D. Hahn , and P. Sureshkumar , “Alarm Interventions for Nocturnal Enuresis in Children,” Cochrane Database of Systematic Reviews 5 (2020): CD002911. 10.1002/14651858.CD002911.pub3 PMC7197139 32364251

[10] S. Schuster , J. Reece , A. Florentzou , and E. Apos , “Treating Enuresis in Children With Neurodevelopmental Disorders Using Bell and Pad Alarm,” Journal of Pediatric Urology 17, no. 5 (2021): 645.e1–645.e8. 10.1016/j.jpurol.2021.07.010 34353751

[11] E. Apos , S. Schuster , J. Reece , et al., “Enuresis Management in Children: Retrospective Clinical Audit of 2861 Cases Treated With Practitioner‐Assisted Bell‐and‐Pad Alarm,” Journal of Pediatrics 193 (2018): 211–216. 10.1016/j.jpeds.2017.09.086 29246468

[12] R. J. Butler and S. L. Gasson , “Enuresis Alarm Treatment,” Scandinavian Journal of Urology and Nephrology 39 (2005): 349–357. 10.1080/00365590500220321 16257835

[13] P. H. Caldwell , M. Lim , and G. Nankivell , “An Interprofessional Approach to Managing Children With Treatment‐Resistant Enuresis: An Educational Review,” Pediatric Nephrology 33 (2018): 1663–1670. 10.1007/s00467-017-3830-1 29110081

[14] M. F. Drummond , M. J. Sculpher , K. Claxton , G. L. Stoddart , and G. W. Torrance , Methods for the Economic Evaluation of Health Care Programmes (Oxford university press, 2015).

[15] Sealed Envelope Ltd , “Power Calculator for Binary Outcome Non‐Inferiority Trial [Internet],” 2012, https://www. sealedenvelope. com/power.

[16] Australia Bureau of Statistics . “Consumer Price Index, Australia Canberra: Australian Government,” 2023, https://www.abs.gov.au/statistics/economy/price‐indexes‐and‐inflation/consumer‐price‐index‐australia/dec‐quarter‐2023.

[17] S. Parab and S. Bhalerao , “Choosing Statistical Test,” International Journal of Ayurveda Research 1, no. 3 (2010): 187–191. 10.4103/0974-7788.72494 PMC2996580 21170214

[18] StataCorp , Stata Statistical Software: Release 18 (StataCorp LLC, 2023).

[19] C. M. Glazener , J. H. Evans , and R. E. Peto , “Alarm Interventions for Nocturnal Enuresis in Children,” Evid.‐Based Child Health 1 (2006): 9–97, 10.1002/ebch.4.

[20] E. Soo and A. Bayne , “Advancements in Treatment Options for Primary Nocturnal Enuresis,” Current Treatment Options in Pediatrics 11, no. 1 (2025): 1–7.

